# Thiolation-enhanced substrate recognition by D-alanyl carrier protein ligase DltA from *Bacillus cereus*

**DOI:** 10.12688/f1000research.4097.1

**Published:** 2014-05-13

**Authors:** Liqin Du, Yu Luo

**Affiliations:** 1 Department of Biochemistry, University of Saskatchewan, Saskatoon, Saskatchewan, S7N 5E5, Canada

## Abstract

D-alanylation of the lipoteichoic acid on Gram-positive cell wall is dependent on *dlt* gene-encoded proteins DltA, DltB, DltC and DltD. The D-alanyl carrier protein ligase DltA, as a remote homolog of acyl-(coenzyme A) (CoA) synthetase, cycles through two active conformations for the catalysis of adenylation and subsequent thiolation of D-alanine (D-Ala). The crystal structure of DltA in the absence of any substrate was observed to have a noticeably more disordered pocket for ATP which would explain why DltA has relatively low affinity for ATP in the absence of any D-alanyl carrier. We have previously enabled the thiolation of D-alanine in the presence of CoA as the mimic of D-alanyl carrier protein DltC which carries a 4’-phosphopantetheine group on a serine residue. Here we show that the resulting Michaelis constants in the presence of saturating CoA for both ATP and D-alanine were reduced more than 10 fold as compared to the values obtained in the absence of CoA. The presence of CoA also made DltA ~100-fold more selective on D-alanine over L-alanine. The CoA-enhanced substrate recognition further implies that the ATP and D-alanine substrates of the adenylation reaction are incorporated when the DltA enzyme cycles through its thiolation conformation.

## Introduction

The cell surface of most Gram-positive bacteria contains wall teichoic acid and lipoteichoic acid with a poly-alditol phosphate backbone. The remaining hydroxyls of the alditol moiety are ubiquitously modified by D-alanyl esterification or glycosylation ^1– 3^. A *dlt* operon, which typically codes for DltA, DltB, DltC and DltD proteins, is required for the D-alanylation of lipoteichoic acids ^4^. D-alanylation brings in positively charged amino-groups and partially neutralizes the net negative charges of the phosphate groups on the lipoteichoic acid backbone. The reduction of D-alanyl content on the cell surface has been found to be associated with increased autolysis ^3, 5^ and susceptibility to host defense peptides and other antibiotics ^6, 7^. Impaired D-alanylation of lipoteichoic acid also reduces the ability of bacteria to colonize any surface ^8^ and form antibiotics-resistant biofilms ^9^. Therefore, the *dlt*-encoded proteins required for the lipoteichoic acid D-alanylation pathway could serve as novel targets for fighting emerging infectious diseases caused by Gram-positive pathogens ^10^. The DltA inhibitor 5’-O-[N-(D-alanyl)-sulfamoyl]adenosine for example, designed by analogy to D-alanyl adenylate (D-Ala-AMP), has been shown to significantly suppress the growth of *B. subtilis* when used in combination with vancomycin ^11^.

**Figure 1.**
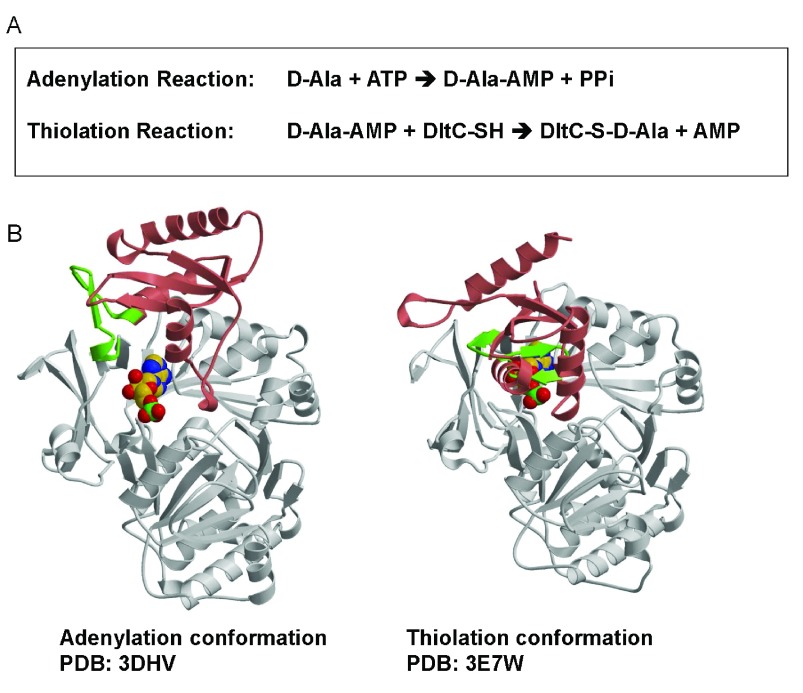
Structure and function of DltA. **A**. A two-step reaction catalyzed by DltA. **B**. The adenylation and thiolation conformations of DltA. In the first adenylation reaction, D-alanine is converted into DltA-bound D-alanine adenylate (D-Ala-AMP). This reaction is catalyzed by the adenylation conformation of DltA shown as DltA/AMP complex. In the subsequent thiolation reaction, the activated alanyl group in the intermediate is transferred to the thiol group of DltC-linked 4’-phosphopantetheine (DltC-SH). The second reaction is catalyzed by the thiolation conformation of DltA shown also as DltA/AMP complex. Most part of the major N-terminal domain of DltA (residues 2 - 399) is shown in gray. The majority of the minor C-terminal domain of DltA (residues 400–504) is shown in salmon. Residues between Arg-397 and Glu-413 are highlighted in green.

The D-alanyl carrier protein ligase DltA (formed by ~500 amino acid residues) ^4^ closely resembles (~30% sequence identity) the adenylation domains (also called adenosine monophosphate (AMP)-forming domains) found in bacterial non-ribosomal peptide synthetases ^12^. Its remote homologs include the acyl- and aryl-(coenzyme A) CoA synthetases and firefly luciferases ^13^. As shown in Figure 1, DltA catalyzes the ATP-driven adenylation of D-alanine and the subsequent transfer of the activated D-alanyl group to the thiol group of 4’-phosphopantetheine, which is covalently bound to a serine side chain of D-alanyl carrier protein DltC (~80 amino acid residues) ^4^. As previously shown for such one-protein-two-enzymes homologs ^14, 15^, crystal structures of two DltA proteins have also been observed in adenylation and thiolation conformations, respectively ^16, 17^ ( Figure 1). The thiolation conformation of *B. subtilis* DltA (BsDltA) ^18^ resembles previously determined structures of a bacterial acetate-CoA synthetase (ACS) and 4-chlorobenzoate-CoA ligase crystallized in their respective thiolation state ^15, 19^. On the other hand, the adenylation conformation of *B. cereus* DltA (BcDltA) ^17^ resembles those observed in the crystal structures of the firefly luciferase PheA (phenylalanine-activating domain of the first module of the *Bacillus brevis* gramicidin S synthetase I), DhbE (2,3-dihydroxybenzoate activation domain from *B. subtilis*), a yeast acetyl-CoA synthetase and 4-chlorobenzoate-CoA ligase ^13, 14, 20– 22^. The two conformations captured in the crystal structures of the two closely related DltA proteins (56% sequence identity) are related by a ~146º rotation around a hinge residue Asp-399 in BcDltA (Asp-398 in BsDltA), which is a invariable residue equivalent to Asp-517 in *Salmonella typhimurium* acetyl-CoA synthetase ^23^. The region surrounding this hinge residue (residues Arg-397 to Glu-413 of BcDltA green in Figure 1B) contains an important triphosphate-interacting residue Arg-397 ^24^ and a β-hairpin which serves as part of the pantetheine channel as observed in the bacterial acetyl-CoA synthetase ^23^.

We previously studied the adenylation reaction of BcDltA in the absence of any D-alanyl carrier to enable the thiolation reaction ^17^. The resulting specificity constants (k _cat_/K _M_) for D- and L-alanine differed by merely ~3-fold, which could not explain the fact that teichoic acid is overwhelmingly modified by D- but not L-alanine ^25^. As suggested by a study on a spectrum of DltA homologs which have residual aminoacyl-CoA synthetase activity ^42^, CoA has been confirmed to be a substitute for the D-alanyl carrier protein DltC as the thiolation substrate of BcDltA ^24^. Here we report further biochemical analysis of BcDltA. Noticeable differences were observed in Michaelis constants K _M_ and turnover rates k _cat_. The presence of CoA, hence the enabled adenylation and thiolation cycle, enhanced the enzyme’s apparent affinities to its cognitive substrate ATP and D-alanine by approximately an order of magnitude. Since BcDltA is a slow enzyme with turnover rate less than 1 s ^-1^, much slower than bacterial Acyl-CoA synthetases (~10 ^2^ s ^-1^) ^23^ and 4-chlorobenzoate-CoA ligase (~10 s ^-1^) ^26^, the observed Michaelis constants should closely approximate the corresponding dissociation constants, and therefore provide some insight into the stability of short-lived enzyme-substrate complexes. We also determined the structure of BcDltA in the absence of any substrate. This structure is noticeably more disordered than previously reported DltA structures ^16, 17, 24^, which may explain the enzyme’s lower affinity to ATP in the absence of the other two substrates. Interestingly, CoA-enhanced affinities to ATP and D-alanine imply that the thiolation substrate CoA-bound BcDltA has higher affinity to both adenylation substrates as compared to CoA-free BcDltA.

## Methods

### Cloning, protein preparation and crystallization

All reagents were from VWR unless specified otherwise. The wild-type as well as the C269A mutant of DltA from *B. cereus* was cloned for over-expression of BcDltA as described previously ^17^. The pET28-BcDltA construct carries an Ala-1 mutation at the N-terminus and eight extra residues at the C-terminus (LEHHHHHH). The soluble fraction of BcDltA was purified by nickel-affinity chromatography followed by gel filtration. BcDltA was concentrated to ~20 mg/mL by ultra-filtration.

### Crystallization and structure determination

The concentrated BcDltA protein was crystallized using the hanging drop crystallization method at a room temperature of 21°C. The optimal well solution for crystallization contained 0.1 M MgCl _2_, 0.5 M KCl, 16% polyethylene glycol (PEG) 3,350 (Sigma-Aldrich) and 0.05 M Hepes-NaOH buffer at pH 7.2. Each drop was composed of 1 μL of protein and 1 μL of well solution. The plate-shaped crystals grew to a maximal size of 0.4 mm × 0.3 mm × 0.05 mm in three days. Crystals were gradually transferred to stabilizing solutions composed of the crystallization well solution supplemented with 8%, 16% and 24% glycerol, soaked for 1 minute, then flash-cooled to - 173.15°C in a nitrogen stream generated by an Oxford CryoSystems device. A total of 400 0.4-degree oscillation images were acquired and processed using a Brukers Proteum-R system as already described ^27^. The previously solved BcDltA model ( PDB code 3DHV) ^17^ was used as the starting model for two-domain rigid body refinement followed by positional refinement using Crystallography & NMR System (CNS) ^28^. This resulting model was subjected to ten cycles of rebuilding and refinement using Arp/Warp ^29^. The rebuilt model was iteratively rebuilt using XtalView ^30^ and then refined using CNS ^28^. The final model had 90.8% of the residues in the most favored regions on a Ramachandran plot. Val-301 with clear electron density and Asp-336 with blurry electron density were the only two residues found in the disfavored region. Statistics of the diffraction data, refinement and geometry are listed in Table 1. The molecular figures were generated using Molscript ^31^ and rendered using Raster3D ^32^. The coordinates and structure factors have been deposited in the Protein Data Bank ^33– 35^ (entry code 4PZP).

**Table 1.** X-ray crystallographic data collection and structure refinement statistics.

	Substrate-free BcDltA
**Data collection**	
Space group	P2 _1_
Cell dimensions	
a, b, c (Å)	52.9, 81.9, 59.3
α, β, γ (°)	90.0, 108.3, 90.0
Resolution (Å)	33.1–1.9 (2.00–1.90) ^a^
^b^R _sym_	0.077 (0.321)
I/σI	7.5 (1.8)
No. reflections	32733 (1982)
Completeness (%)	86.3 (37.7)
Redundancy	4.3 (3.2)
**Refinement**	
Resolution (Å)	30.0–1.9
No. reflections	32658
^c^R _work_/R _free_	0.218/0.261
No. atoms:	
Protein	3643
Water	279
Average B-factors:	
Protein	22.9
Water	27.7
R.m.s. deviations	
Bond lengths (Å)	0.009
Bond angles (°)	1.47

Notes:

^a^ Values in parentheses refer to values in the highest resolution shell.

^b^ R _sym_ = Σ| I _h_ - <I> _h_ |/ΣI _h_, where <I> _h_ is average intensity over symmetry equivalents, h is reflection index. The summation is over all measured reflections.

^c^ R _work_ = Σ| F _calc_ - F _obs_ |/ΣF _obs_. The summation is over all reflections used in refinement. R _free_ is calculated using a randomly selected 5% of the reflections set aside throughout the refinement.

### Tryptophan fluorescence measurement

The intrinsic tryptophan fluorescence of 1.0 ml 0.4 uM BcDltA solution with 0 to 2 mM ATP was acquired at a room temperature of 21°C using a PerkinElmer LS-55 fluorescence spectrometer. The excitation wavelength was 305 nm and a fluorescent emission in the 310 nm to 390 nm range was recorded. The relative fluorescence increase at 345 nm was used to quantify the ATP-bound fraction of BcDltA. Assuming that the fluorescence gain is proportional to [ATP]/(K _D_ + [ATP]), Prism software (GraphPad Software) was used to derive the dissociation constant of ATP.

### Pyrophosphate quantification assay

As previously described, pyrophosphate released from the adenylation reaction is broken down into phosphate by pyrophosphatase ^17^. The resulting phosphate was quantified by a dye solution containing 0.033% w/v Malachite Green, 1.3% w/v ammonium molybdate and 1.0 M HCl ^36^. The 200 μL reaction solutions contained 5 μM BcDltA, 0.1 M KCl, 0.01 M MgCl _2_, 0.05 M Tris-Hepes buffer at pH 7.2, 5 unit/mL of inorganic pyrophosphatase from baker’s yeast (Sigma-Aldrich) and specified concentrations of D-alanine, ATP and CoA (Sigma-Aldrich). A volume of 25 μL of reaction solution was retrieved every 3 or 5 minutes and mixed thoroughly with 475 μL of the dye solution. The absorption at a wavelength of 620 nm was recorded after 90 seconds. The initial rates (1/2 of the phosphate concentration increase per minute) of the adenylation reaction were derived from the time courses of phosphate accumulation. The correlation between initial reaction rate and substrate concentration was fitted with Michaelis-Menten equation using the Prism software (GraphPad Software).

### Thiol quantification assay

The free thiol group of CoA was quantified as described previously ^24^ by a dye solution composed of 1 mM 5,5’-dithio-bis (2-nitrobenzoic acid) (DTBN), or Ellman’s reagent (Sigma-Aldrich) and 50 mM Tris-EDTA solution at pH 8.0. Absorption at a wavelength of 412 nm was used to quantify the concentration of free thiol group. The reaction rate was derived by the rate of thiol depletion. The correlation between initial reaction rate and substrate concentration was also fitted with Michaelis-Menten equation using the Prism software.

## Results

### Crystal structure of substrate-free BcDltA

**Figure 2.**
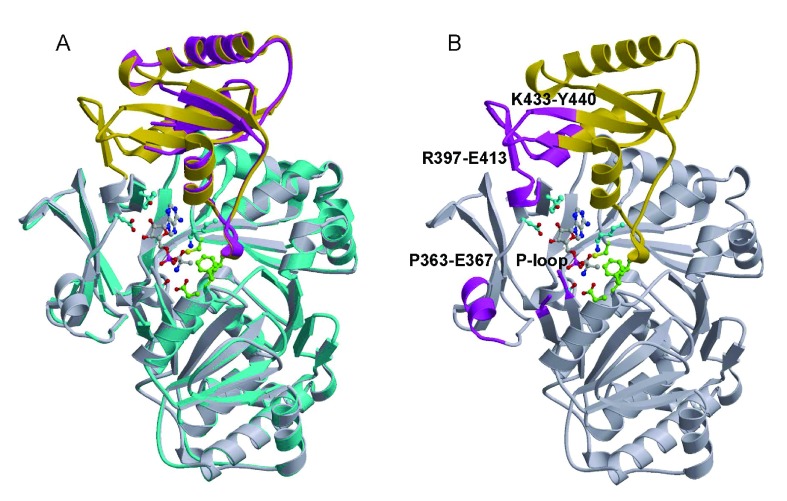
Structure of DltA in the absence of substrate. The ribbons representation of previously reported BcDltA structure (PDB entry: 3DHV) is shown with D-alanine-adenylate and surrounding side-chains in ball-and-stick model. The N-terminal domain is shown in gray, and the C-terminal domain in gold. **A**. The substrate-free structure of BcDltA is superimposed on the BcDltA/D-Ala-AMP complex. The N- and C-terminal domains are colored in cyan and magenta, respectively. **B**. The four corresponding regions of the BcDltA/D-Ala-AMP complex, which are disordered in the substrate-free form of BcDltA, are highlighted in magenta.

The same DltA protein from *B. cereus* with a C-terminal hexahistidyl fusion tag used in our previous crystallographic studies on BcDltA ^17, 24^ was crystallized in the absence of ATP, D-alanine or CoA. One crystal diffracted to 1.9 Å resolution and belonged to space group P2 _1_ ( Table 1), the same space group as in previously reported crystals of BcDltA in complex with D-alanine adenylate ^17^ and with ATP ^24^. Despite having 8 Å shorter crystallographic a axis, 5 Å shorter b axis and 5° smaller β angle than the previously reported crystal of DltA/D-alanine adenylate complex, the structure was successfully solved by rigid-body refinement using the previously determined BcDltA structure ^17^ ( PDB code 3DHV) as the starting model. The 504-residue BcDltA structure can be divided into two domains ( Figure 2): an N-terminal major domain from the N-terminus to Asp-399, and a C-terminal minor domain from residue 400 to the C-terminus. The disposition of the two domains in the substrate-free BcDltA structure remains similar to that of the starting model ( Figure 2A). The electron density map indicated several disordered regions (Ser-153 to Pro-159, Pro-363 to Glu-367, Arg-397 to Glu-413, Lys-433 to Tyr-440) with the corresponding regions in the starting model highlighted in magenta in Figure 2B. The first disordered region is part of a highly conserved P-loop (Thr-152 to Lys-160) found in homologous AMP-forming proteins ^37– 39^. Due to its similar amino acid composition (glycine, serine, threonine and lysine) to that of P-loop or Walker A motif found in ATPases and GTPases ^40^, this loop has long been thought to catalyze the adenylation reaction. In the crystal structure of human medium-chain acyl-CoA synthetase in complex with ATP, this loop intimately interacts with the β- and γ-phosphates of the ATP substrate ^41^. Functional relevance of regions 363–367 and 433–440 are unknown. The former is located at a 2-turn helix at the surface of the N-terminal domain ( Figure 2B). The latter is interacting with the longest disordered inter-domain region in this structure (397–413) which contains several key elements of DltA. Arg-397 has been observed to interact with the β-phosphate of ATP and to play an important role in catalysis ^24^. Asp-399, equivalent to Asp-398 in BsDltA, serves as the hinge residue for domain rotation. As observed for the equivalent Asp-402 of 4-Chlorobenzoate-CoA ligase ^15^, the rotation around main-chain single bonds in this hinge residue could account for a 146° swing of the C-terminal domain as we compared the crystallized adenylation conformation of BcDltA and thiolation domain of BsDltA ( Figure 1, bottom with the disordered inter-domain linker in green). The equivalent main-chain atoms in the N-terminal domains of BcDltA in adenylation conformation ( PDB entry 3DHV) and BsDltA in thiolation conformation ( PDB entry 3E7W) are superposed with a root-mean-deviation of 0.97 Å, and the deviation for the C-terminal domain was 1.00 Å. These values indicate that there is no dramatic conformational change within each domain in addition to the 146° rotation around the hinge aspartate residue. The C-terminal part of this flexible inter-domain region also contains a β-hairpin which has been observed to interact with CoA in homologous acetyl-CoA synthetase ^23^.

### K _M_ and k _cat_ of BcDltA in the presence and absence of D-alanyl carrier CoA

**Figure 3.**
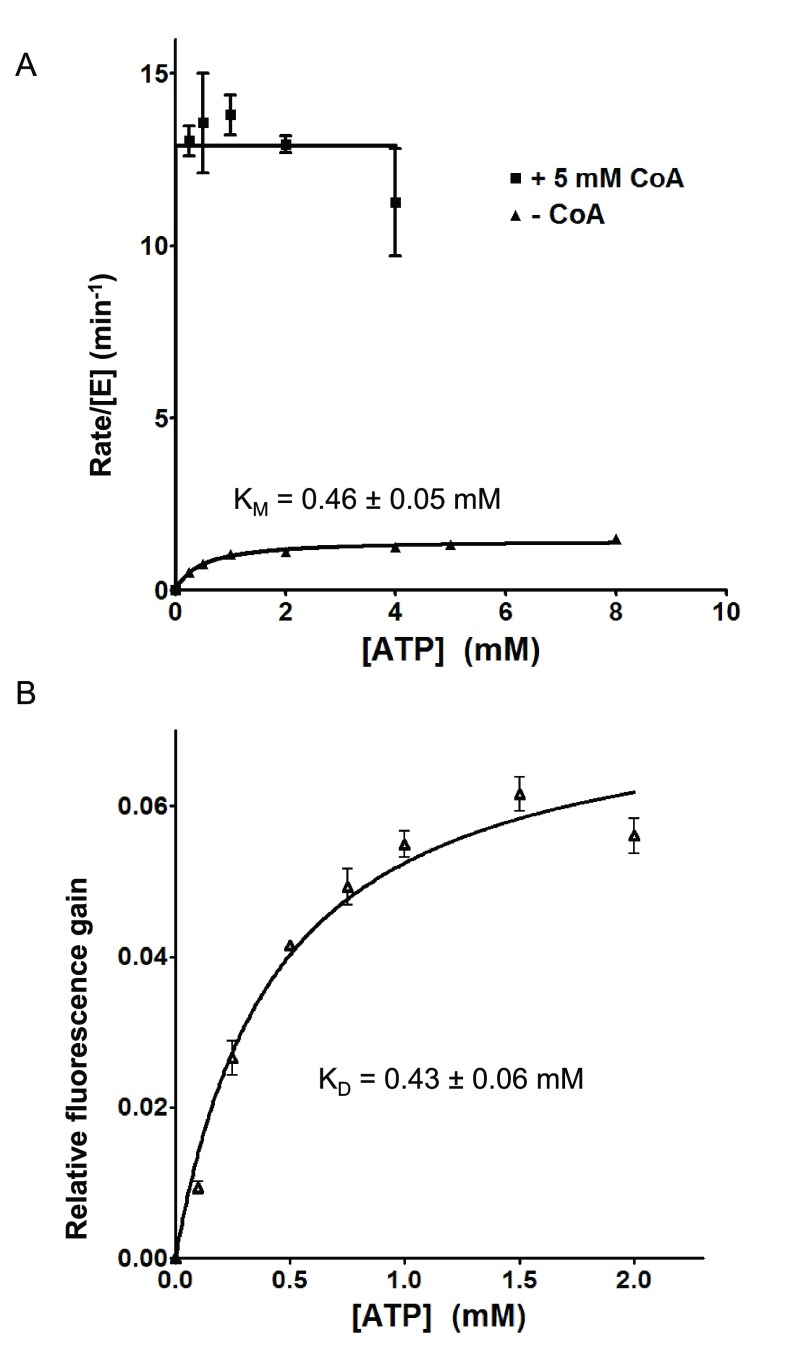
Rate of pyrophosphate release and ATP-induced fluorescence gain. The reaction solutions contained 0.005 mM wild-type BcDltA, specified concentrations of ATP and CoA, 5 mM D-alanine, 0.1 M KCl, 0.01 M MgCl _2_, 0.05 M Hepes-NaOH buffer at pH 7.2, 5 unit/mL of yeast inorganic pyrophosphatase. **A**. The initial rates of pyrophosphate accumulation divided by the BcDltA concentration are shown. The reaction rates in the presence of CoA are taken from a previous study ^24^. **B**. Relative fluorescence gains as a fraction of the fluorescence intensity in the absence of ATP are shown.

**Figure 4.**
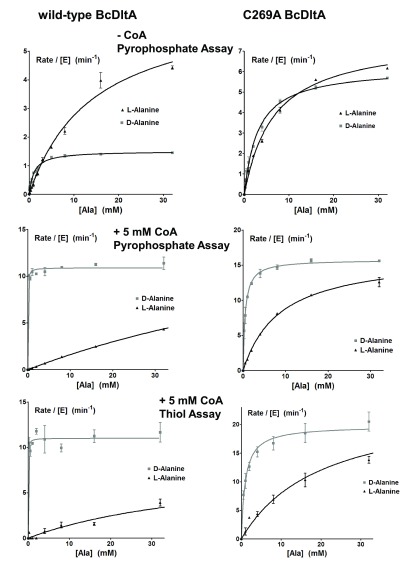
Rate of pyrophosphate release and thiol depletion. The reaction solutions contained 0.005 mM wild-type BcDltA, 5 mM D-alanine, 0.1 M KCl, 0.01 M MgCl _2_, 0.05 M Hepes-NaOH buffer at pH 7.2, 5 unit/mL of yeast inorganic pyrophosphatase and specified concentrations of ATP, alanine and CoA. Reaction rates for wild-type BcDltA are shown on the left, and those for the C269A mutant protein are shown on the right. The reaction rates in the absence of CoA are taken from a previous study ^17^.

In our previous study, we have verified that CoA can mimic D-alanyl carrier protein DltC ^24^, as also discovered for DltA homologs ^42^. In that study, we have observed that the reaction rate is increased by nearly an order of magnitude by the presence of saturating concentration of CoA, which is explained by the faster release of the thiolation product rather than by release of the adenylation intermediate. In order to get a comprehensive understanding of the effects by CoA as the DltC mimic, we further studied the enzymatic properties of BcDltA in the presence of a saturating concentration (5 mM) of ATP or D-alanine, and in the absence of CoA or in the presence of a saturating concentration (5 mM) of CoA. The reaction rates derived from the pyrophosphate accumulation assay and the thiol depletion assay were similar ( Figure 4). The thiolation assay was noticeably noisier than the pyrophosphate assay and we therefore limit the discussion to K _M_ and k _cat_ values derived from the pyrophosphate assay. Somewhat unexpectedly, BcDltA showed much higher apparent affinity, or decreased K _M_ value, towards ATP (0.46 mM to 0.01 mM) and D-alanine (1.1 mM to 0.03 mM) in the presence of 5 mM CoA ( Figure 3 and Figure 4, Table 2). On the contrary, the apparent affinity towards L-alanine decreased in the presence of CoA, with K _M_ increased from 14.4 mM to 109 mM ( Figure 4 and Table 2).

**Table 2.** Kinetic data for DltA protein from *B. cereus*.

BcDltA (substrate)	K _M_ (× 10 ^-3^ M)	k _cat_ (min ^-1^)	k _cat_/K _M_ (× 10 ^3^ M ^-1^·min ^-1^)
**Pyrophosphate assay (5 mM ATP, - CoA) (previous work ^17^)**			
Wild-type (D-Ala)	1.1 ± 0.2	1.5 ± 0.1	1.4 ± 0.2
Wild-type (L-Ala)	14.4 ± 1.6	6.7 ± 0.4	0.47 ± 0.05
C269A (D-Ala)	3.1 ± 0.3	6.2 ± 0.2	2.0 ± 0.2
C269A (L-Ala)	6.6 ± 0.5	7.6 ± 0.3	1.2 ± 0.1
**Pyrophosphate assay (5 mM ATP, 5 mM CoA) (this work)**			
Wild-type (D-Ala)	0.03 ± 0.01	10.9 ± 0.2	363 ± 100
Wild-type (L-Ala)	109 ± 7	19.3 ± 0.7	0.18 ± 0.02
C269A (D-Ala)	0.50 ± 0.05	15.8 ± 0.3	32 ± 3
C269A (L-Ala)	8.8 ± 0.5	16.5 ± 0.4	1.9 ± 0.2
**Thiol assay (5 mM ATP, 5 mM CoA) (this work)**			
Wild-type (D-Ala)	0.02 ± 0.02	11.0 ± 0.3	550 ± 200
Wild-type (L-Ala)	48 ± 12	8.4 ± 1.0	0.18 ± 0.02
C269A (D-Ala)	1.0 ± 0.2	19.8 ± 0.8	19.8 ± 0.9
C269A (L-Ala)	20 ± 3	24.3 ± 1.6	1.2 ± 0.2
**Pyrophosphate assay (5 mM D-Ala, 5 mM CoA) (previous work ^24^)**			
Wild-type (ATP)	0.01 ± 0.02	12.9 ± 0.6	1290 ± 2000
**Pyrophosphate assay (5 mM D-Ala, - CoA) (this work)**			
Wild-type (ATP)	0.46 ± 0.05	1.46 ± 0.04	3.2 ± 0.04

### Relaxed D-alanine preference by the C269A BcDltA mutant protein

The side-chain of Cys-269 sits at the bottom of the D-alanine-binding pocket which may make VDW clash with the methyl side-chain of L-Alanine ^16, 17^. We also studied the effect of CoA on D- and L-alanine preference of the C269A mutant of BcDltA ( Figure 4 and Table 2). In the presence and absence of CoA, the C269A protein showed relaxed preference for D-alanine over L-alanine. As observed for the wild-type protein, CoA also enhances the D-alanine preference of this BcDltA mutant protein. However, the CoA-induced changes in Michaelis constant K _M_ were less dramatic (3.1 mM to 0.50 mM for D-alanine, 6.6 mM to 8.8 mM for L-alanine) than those observed for the wild-type protein.

### ATP binding by BcDltA

Change in tryptophan fluorescence of BcDltA was minimal in the presence of ATP in the 10 micromolar range. We then found that at an excitation wavelength of 305 nm, the absorption of up to 2 mM of ATP was negligible and there was fluorescence gain associated with increasing concentration of ATP. The fluorescence gain-derived dissociation constant K _D_ for ATP (0.43 mM) was similar to the Michaelis constant K _M_ (0.46 mM) derived in the absence of CoA ( Figure 3). There was no detectable fluorescence change to derive K _D_ values for D-alanine. For CoA, we were not able to isolate fluorescence change from absorption by CoA in millimolar concentration.

Data of pyrophosphate release, ATP-induced fluorescence and thiol depletion for DltA in Bacillus cereus Data file 1 A phosphate accumulation assay is shown: rate dependence on ATP concentration (repeats both with 5 mM CoA and mM D-Ala). Data file 2 A phosphate accumulation assay is shown: rate dependence on ATP concentration (repeats both without CoA but with 5 mM D-Ala). Data file 3 Data of fluorescence gain versus ATP concentration are shown (relative fluorescence gain for 4 repeats). The excitation wavelength was 305 nm. The emission wavelength was 345 nm. Data file 4 A phosphate accumulation assay is shown for the wild type BcDltA: rate versus [Alanine] in the absence of CoA, + 5 mM ATP. Data file 5 A phosphate accumulation assay is shown for the mutant C269ABcDltA: rate versus [Alanine] in the absence of CoA, + 5 mM ATP. Data file 6 A phosphate accumulation assay is shown for the wild type BcDltA: rate [Alanine] in the presence of 5 mM CoA, + 5 mM ATP. These data were generated with a different batch of protein at a very high L-Ala concentration. Data file 7 A thiol depletion assay is shown for the wild type BcDltA: rate versus [Alanine] in the presence of 5 mM CoA, + 5 mM ATP. Data file 8 A thiol depletion assay is shown the mutant C269ABcDltA: rate versus [Alanine] in the presence of 5 mM CoA, + 5 mM ATP

## Discussion

### DltA strongly prefers D- over L-alanine

Bacteria selectively incorporate D- over L-alanine in cell wall components. There appears to be no exception in ubiquitous esterification of lipoteichoic acids by alanine ^25^. The enantiomer selectivity of BcDltA observed in the absence of any D-alanyl carrier ^17^, however, has been intriguingly mediocre. The newly acquired kinetic data in the presence of saturating CoA, a DltC mimic, shows that the k _cat_ values are less than 2-fold different for D- and L-alanine (10.9 and 19.3 min ^-1^) while the K _M_ values are more than 1000-fold different favouring D-alanine over L-alanine (0.03 and 109 mM). Adding the fact that the intracellular concentration of D-alanine (in the order of 10 ^2^ μM) is approximately 10-fold more abundant than the L-enantiomer (in the order of 10 ^1^ μM) ^43^, DltA functioning at a saturating concentration of the D-alanyl carrier protein DltC would favor the ligation of the D-enantiomer by approximately 4 orders of magnitude. Such striking enantiomer selectivity is consistent with the much lower L-alanine content found in lipoteichoic acid ^25^. A recent study has shown that the *dlt* operon is induced by cell envelope stress such as acidic pH and antibiotics ^44^. It is possible that stress-induced expression of DltC may reach a saturating concentration for interacting with DltA and therefore ensure the almost exclusive enantiomer selectivity as observed in the presence of the DltC mimic. The comparison between the kinetic properties of the wild-type and the C269A mutant proteins also supports the notion that Cys-269, and its equivalence in other DltA proteins, contributes to the enantiomer selectivity of DltA ^16, 17^.

### Thiolation conformation of DltA is compatible with adenylation substrates

It is satisfying to observe the stringent D-alanine preference. At the same time, it is also puzzling to appreciate that such selectivity on the chirality of alanine, a substrate of the adenylation reaction, can only be achieved in the presence of CoA, a substrate of the thiolation reaction. The intrinsic fluorescence of tryptophan in BcDltA enabled us to derive the dissociation constant K _D_ for ATP in the absence of other substrates. The new form of crystal structure in the absence of any substrate is noticeably more disordered than the previously observed adenylation conformation of BcDltA ^17^ and thiolation conformation of BsDltA ^16^. The longest such disordered region is between Arg-397 and Glu-413, which contains the inter-domain hinge residue Asp-399, interacts with β-phosphate of ATP, and forms the pantetheine channel. Possibly, this important region remains disordered in the presence of saturating D-alanine, therefore providing an explanation for the similar values between the above mentioned K _D_ (0.43 mM) and the K _M_ (0.46 mM) for ATP, in the presence of saturating D-alanine but in absence of CoA. The more disordered nature of the substrate-free conformation of BcDltA also implies that the previously observed adenylation and thiolation conformations are intrinsically unstable unless stabilized by the interaction with one or more substrates. This structural feature of BcDltA likely explains the relatively low sub-millimolar affinity for ATP, since part of the stabilizing BcDltA-ATP interactions would be used to compensate the cost of establishing the adenylation conformation of the protein.

BcDltA is a very slow enzyme. Unless the substrate dissociation step happens to be extremely slow as well, we could approximate the observed K _M_ values to the K _D_ values of corresponding BcDltA-substrate intermediates, and therefore enable reasoning in the context of structural stability of such intermediates. As such, we reasoned that the approximately one order of magnitude difference in K _M_ values for the adenylation substrates ATP and D-alanine observed in the presence and absence of the thiolation substrate may imply the existence of a quadruple intermediate of the DltA enzyme in complex with all three substrates, which may be markedly different from a ternary intermediate of BcDltA with the two adenylation substrates.

**Figure 5.**
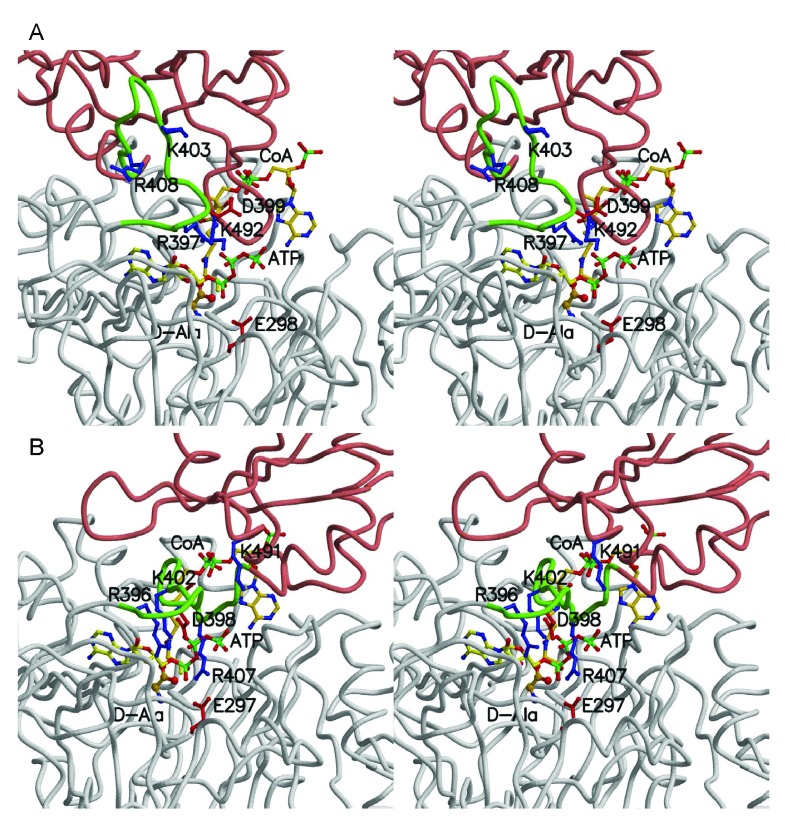
Structural model of DltA in complex with ATP, D-alanine and CoA. Cα traces of the two modelled quadruple complexes are shown in stereo. Except for the green-colored region between Arg-397 and Glu-413, the N- and C-terminal domains are colored in gray and salmon, respectively. The three substrates and selected side-chains are shown in ball-and-stick model. **A**. The quadruple complex in adenylation conformation. Residue numbers in BcDltA are shown. **B**. The quadruple complex in thiolation conformation. Residue numbers in BsDltA are shown.

We then resorted to three-dimensional model building so as to answer the question on which of the adenylation and thiolation conformations may be compatible with binding to all three substrates. The BcDltA/D-alanine-adenylate complex ( PDB entry 3DHV) ^17^ was chosen as the adenylation conformation and as the reference set of atomic coordinates. The adenylated intermediate was dissected to generate the D-alanine model. The N-terminal domain of the BcDltA/ATP complex ( PDB entry 3FCE) ^24^, which is also in the adenylation conformation, was superposed on the reference set to orient the ATP substrate. The N-terminal domain of the BsDltA/AMP complex (PDB entry 3E7W) was also superposed on the reference set to derive the re-oriented thiolation conformation. Main-chain atoms equivalent to those interacting with AMP in BcDltA (270–272, 292–299) in the quadruple complex of acetyl-CoA synthetase in its thiolation conformation (PDB entry 2P2F) ^23^ were superimposed on the reference set to orient the CoA model. In the adenylation conformation ( Figure 5A, the pantetheine channel is apparently blocked by the main-chain atoms immediately preceding the catalytic Lys-492 of BcDltA (Lys-491 of BsDltA). Although we could not completely rule out the possibility that an allosteric site for CoA exists, no such site has ever been observed for this superfamily of enzymes. Therefore, a quadruple complex in the adenylation conformation is unlikely. The thiolation conformation ( Figure 5B), on the other hand, appears to be compatible with binding ATP so long as Arg-408 of BcDltA (Arg-407 of BsDltA) adopts another rotamer. It has been previously observed that the homologous acetyl-CoA synthetase in its thiolation conformation binds AMP, acetate and CoA ^23^, and that BsDltA in its thiolation conformation binds AMP and appears to have a well-formed D-alanine-binding pocket ^16^. These structural evidences seem to suggest that a quadruple intermediate may form with the enzyme in its thiolation conformation but not in the adenylation conformation. Since the thiolation conformation has an AMP-binding site and its N-terminal domain, which provides most of the ATP-interacting residues such as the P-loop and Arg-397, remains essentially identical to that that in the adenylation conformation, it is not surprising that only one arginine side-chain is required to adopt another rotamer to accommodate ATP. This arginine residue (Arg-408 of BcDltA) forms a salt bridge with the divalent cation-anchoring side-chain of Glu-298 in BcDltA (Glu-297 in BsDltA) ^24^, which appears to be an important structural feature to modulate the conformational change ^16^. Another arginine residue (Arg-397 of BcDltA, Arg-396 of BsDltA) also seems to facilitate the conformational change. It forms a salt bridge with the hinge aspartate residue in the thiolation conformation. In the adenylation conformation, this arginine side-chain adopts a more extended rotamer and forms a salt bridge with the β-phosphate group of ATP ( Figure 5A). ATP binding would result in re-orientation of both arginine residues and disruption of two salt bridges, thus mobilizing the thiolation conformation.

### Structural basis for CoA-enhanced affinity for ATP

The majority of ATP-binding elements lie in the N-terminal domain which remains similar in both adenylation and thiolation conformations. The most significant structural feature in the C-terminal domain for binding ATP is the catalytic Lys-492 of BcDltA (Lys-491 of BsDltA) in the adenylation conformation. In the thiolation conformation, the catalytic residue is replaced by Lys-403 (Lys-402 of BsDltA) ( Figure 5). In addition, the thiolation conformation is more ordered than the substrate-free conformation. The favourable DltA/ATP interactions may no longer be used to compensate the energetic cost of stabilizing the disordered hinge region. Therefore the affinity for ATP by the CoA-bound BcDltA in its thiolation conformation should be higher than substrate-free BcDltA.

### Structural basis for CoA-enhanced enantiomer selectivity for D-alanine

For both the wild-type and C269A mutant BcDltA, the presence of saturating CoA increases the apparent affinity for D-alanine while decreasing affinity for L-alanine. The majority of alanine-interacting residues lie in the N-terminal domain. The amino group of D-alanine is stabilized by Asp-197 of BcDltA (Asp-196 of BsDltA). Cys-269 of BcDltA (Cys-268 of BsDltA) lies close to Cα of D-alanine and serves as one major determinant of enantiomer selectivity. The carboxylate group of D-alanine is stabilized by Lys-492 in the adenylation conformation or by Lys-403 in the thiolation conformation. At the end of the pantetheine channel, Phe-196 of BcDltA (Phe-195 of BsDltA) adopts different rotamers in the two conformations. In the adenylation conformation of BcDltA/D-alanine adenylate, the shortest distance from Phe-196 side-chain to methyl group of D-alanine is 4.15 Å. In the modelled quadruple complex in the thiolation conformation, the Phe-196 side-chain does not contact D-alanine but the thiol group of CoA lies in closer proximity of D-alanine side-chain (3.34 Å), as expected from the pending thiolation reaction between the thiol group of CoA and the carbonyl group of D-alanine-adenylate. On the other hand, the tighter alanine-binding pocket may exert stronger VDW repulsion toward L-alanine, thus lowering further the affinity for the wrong enantiomer. Interestingly, Cys-269 also has a thiol group. The removal of this group in the C269A mutant of BcDltA reduces the affinity for D-alanine and increases the affinity for L-alanine by approximately one order of magnitude, which is almost exactly the opposite to the effect of introducing the thiol group of CoA in the alanine-binding pocket. Since sulphur atoms are larger and more inducible than the second-period elements carbon and oxygen, it is not surprising that the VDW interaction involving a thiol group makes a significant impact on the stability of enzyme/substrate complex.

### Hypothesized enzymatic cycle of DltA

**Figure 6.**
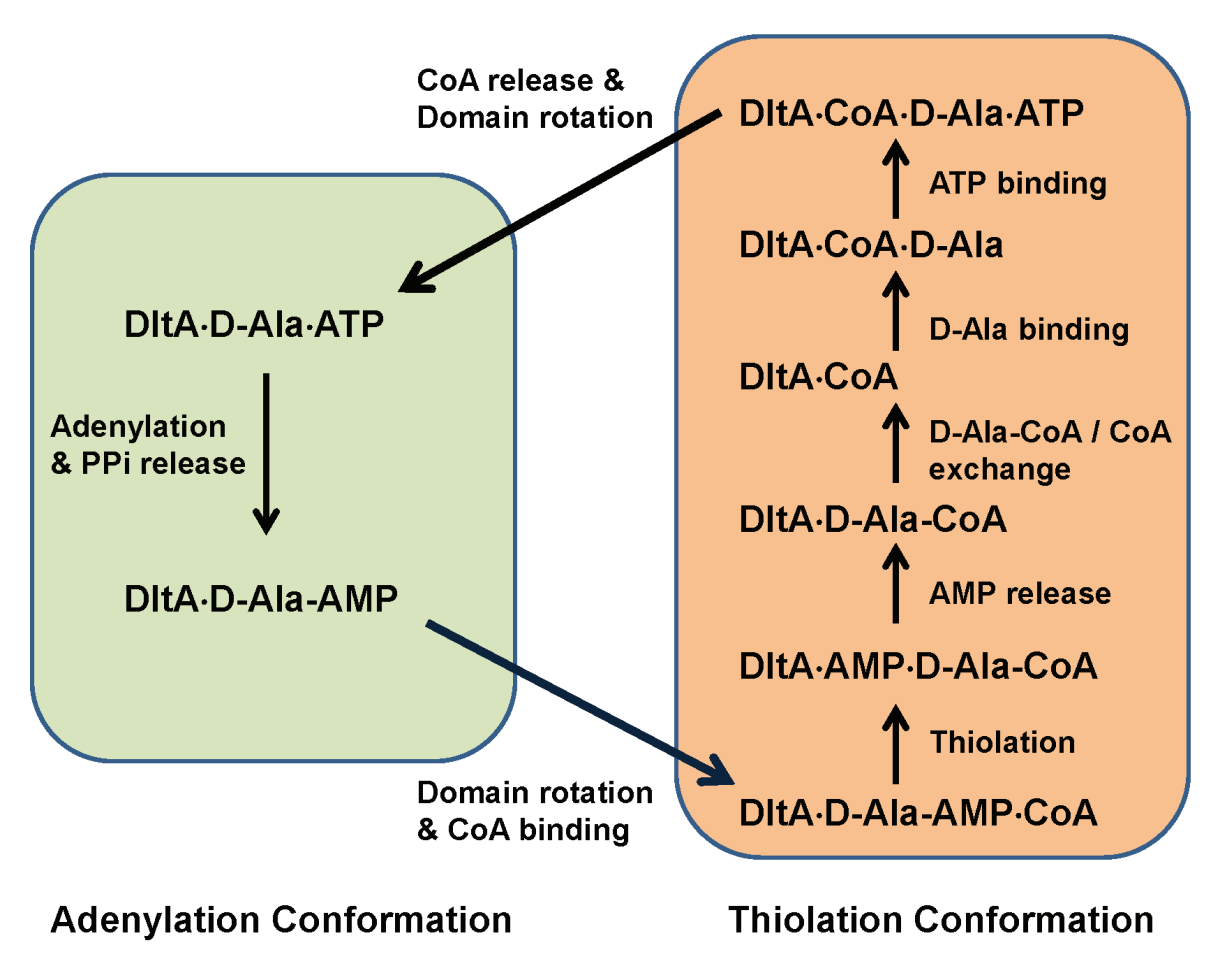
Hypothetical enzymatic cycle of DltA. Non-covalent DltA complexes are shown in text. Forward reaction, substrate binding and release steps are shown by arrows. Steps fulfilled by the adenylation conformation are shown in the green box, and those by the thiolation conformation in brown. The thioester between D-Ala and CoA (D-Ala-CoA) is the final product.

Intracellular concentration of D-alanine ^43^ generally exceeds the K _M_ value in the presence of saturating ATP and CoA. Typical intracellular concentration of ATP lies in the millimolar range ^45^, exceeding the K _M_ value for ATP as well. The concentration of the possibly stress-induced DltC ^44^ may also reach saturating level in bacteria when such stress is present. As implied by the CoA-triggered dramatic change in K _M_ values for ATP and D-alanine, the two adenylation substrates are likely incorporated by the enzyme with pre-bound CoA rather than with merely the other adenylation substrate. In addition, three-dimensional modelling suggests the CoA-bound state of the enzyme can only exist in its thiolation conformation. We therefore hypothesize a two-conformation model for the enzymatic cycle catalyzed by DltA ( Figure 6) in the presence of saturating concentration of the three substrates. Our model differs from the three-conformation model proposed for DltA and adenylation domains in non-ribosomal peptide synthetase ^16^ which includes a third substrate-free conformation. This model also differs from the two-conformation model proposed for and for 4-chlorobenzoate-CoA ligase ^26, 46^ which includes substrate-free state of the enzyme. The two previously proposed models are consistent with a typical Ping-Pong mechanism. Neither a substrate-free conformation nor a substrate-free state of the enzyme is required in our model for the enzymatic cycle of DltA once the protein enters the reaction cycle. The adenylation reaction starts from a ternary complex with ATP and D-alanine and proceeds with the release of pyrophosphate. A domain rotation around the aspartate hinge residue follows transforming into the thiolation conformation which binds CoA, or DltC in bacteria, and catalyzes the thiolation reaction with D-alanine by displacing AMP. In our model, the resulting complex with the two thiolation products proceeds with AMP release, D-alanine-CoA/CoA exchange, D-alanine binding, and ATP binding. In the quadruple complex, the ATP-bound magnesium ion would replace Arg-408 of BcDltA in forming a bridge with Glu-298, as observed in the crystal structure BcDltA/ATP complex ^24^. The disruption of the Arg-408 to Glu-298 salt bridge would destabilize the thiolation conformation and facilitate a reverse domain rotation and release of CoA which is not compatible with the adenylation conformation. The hypothesized D-alanine-CoA/CoA exchange step is the central piece of this enzymatic cycle, which reflects the finding that CoA affects DltA’s apparent affinities with D-alanine and ATP. If DltA were to become substrate-free for a long enough period of time, it would form a ternary complex with ATP and D-alanine, which contradicts the observed effect by the thiolation substrate CoA. It is worth noting that the sequence of D-alanine and ATP binding is uncertain. Similarly, AMP release has to occur before ATP binding but not necessarily before exchange with CoA or D-alanine binding.

The hypothesized enzymatic cycle involves a second CoA binding step, and a CoA release step in addition to the Ping-Pong mechanism previously proposed for DltA and its homologs. Both additional steps seem unnecessary for the enzymatic reaction itself, but are required to explain our enzymatic data and are consistent with the three-dimensional models of BcDltA and BsDltA. A Ping-Pong mechanism would require that the binding of CoA and the binding of either adenylation substrate be uncompetitive, and therefore the apparent K _M_ and k _cat_ values for either adenylation substrate both would become larger at higher CoA concentration. While the k _cat_ values for both ATP and D-alanine did become larger at saturating CoA concentration, the K _M_ values actually became smaller, therefore contradicting the typical Ping-Pong mechanism. Another property of aminoacyl-CoA synthetases including DltA is the inhibitory effect of CoA at high concentration ^42^, which is difficult to explain by a typical Ping-Pong mechanism unless the enzymatic cycle includes an additional CoA-dissociation step as in our model. The closest homologs of DltA are amino acid-activation domains found in non-ribosomal peptide synthetases ^42^. Similar to DltA, these homologs also pass the adenylated intermediate to the 4’-phosphopantetheine group attached to a serine residue on a peptide carrier domain. It is possible that such amino acid activation domains may also act as DltA.

The extra binding step for 4’-phosphopantetheine D-alanyl carrier could serve as the sensor for the availability of the carrier. The maximum rate catalyzed by BcDltA is approximately seven times faster in the presence of CoA than in its absence, with respective k _cat_ values 10.9 min ^-1^ and 1.5 min ^-1^. Moreover, the intracellular concentration of D-alanine is typically found in the 100 micromolar range ^43^, which lies above the K _M_ value for D-alanine in the presence of CoA (~30 µM) but below the K _M_ value in the absence of CoA (1100 µM) ( Table 2). The reaction rate should become slower by approximately 100 fold when the thiol carrier is absent. It is worth noting that the adenylated D-alanine intermediate generated by the adenylation reaction is not covalently attached to the enzyme and could be released and wasted when the thiolation substrate is absent. The significant slowing down of the adenylation reaction in the absence of the 4’-phosphopantetheine carrier therefore provides a biological advantage.

## Data availability

*figshare*: Data of pyrophosphate release, ATP-induced fluorescence and thiol depletion for DltA in *Bacillus cereus*, http://dx.doi.org/10.6084/m9.figshare.1018489 ^47^

## Supplementary file

PDB validation report
