# Supplementary material for: Thiolation-enhanced substrate recognition by D-alanyl carrier protein ligase DltA from Bacillus cereus
Source: F1000Res. 2014 May 13;3:106. [Version 1] doi: 10.12688/f1000research.4097.1 (PMC4176424; doi:10.12688/f1000research.4097.1)
Supplement: Peer review history

# Peer review: Referee response for version 1

**DOI:** 10.5256/f1000research.4389.r5026

**Published:** 2014-06-17

**Authors:** Charles Bell

**Affiliations:** 1 Department of Chemistry and Biochemistry, Ohio State University, Columbus, OH, USA

ltA catalyzes two reactions: (1) an adenylation reaction in which ATP is joined to D-Ala to form D-Ala-AMP (PPi is released), and (2) a thiolation reaction in which D-Ala is transferred from D-Ala-AMP to a phosphopantetheine group of D-alanyl carrier protein (DltC). The reaction can be mimicked in vitro by substituting CoA for DltC (does this also happen in vivo?). Previous crystal structures have indicated that the two reactions are promoted by two distinct conformational states of the enzyme, which differ by a large rearrangement of a C-terminal domain that closes over the active site. In this work, Du & Luo provide kinetic data to suggest that binding of the two substrates for the adenylation reaction (ATP and D-Ala) is promoted by the presence of the substrate (CoA) for the second reaction. The selectivity for binding of D-Ala as opposed to L-Ala is also enhanced significantly by the presence of CoA. A new scheme for the reaction cycle is proposed in which instead of forming an empty enzyme when the final product D-Ala-CoA is released, free CoA is rapidly “exchanged” in for D-Ala-CoA to keep the enzyme in its thiolation conformation. Then new D-Ala and ATP substrates bind (with the enzyme still in its CoA-bound thiolation conformation), and CoA is released to allow the enzyme to adopt the adenylation conformation, which is required for ATP and D-Ala to react. Binding of CoA to the newly formed D-Ala-AMP complex then puts the enzyme back in the thiolation conformation. The new reaction scheme is considerably more complicated than one would expect, but seems to account for the observed kinetic data. The authors propose that the biological purpose for this type of mechanism is to limit binding (and reaction) of ATP and D-Ala to conditions in which the 2 ^nd^ substrate (presumably DltC in vivo) is available, so as to avoid non-productive formation and release D-Ala-AMP.

A crystal structure of the apo form of DltA is also determined, which shows that in the absence of substrates the enzyme adopts the adenylation conformation, and that several loops on the enzyme are considerably more disordered than in previous structures of various substrate or product-bound states. The new structure does not add a lot, but is meant to support the concept that ATP and D-Ala do not bind tightly to the enzyme unless CoA is around to reduce the overall flexibility.

The manuscript is reasonably well written, scientifically sound, and should be of fairly broad interest, particularly for those studying other types of two-reaction enzymes, such as in non-ribosomal peptide synthesis, and possibly even enzymes like amino-acyl tRNA synthetases. I found the manuscript to be rather uninteresting, until I got to the end of the discussion, where the newly proposed reaction scheme of Figure 6 is presented, and a biological purpose of the observed phenomena is proposed. If there is some way to bring these aspects into the Abstract or the Introduction, a potential reader might be drawn in a little bit earlier than I was.

The proposed scheme is quite unusual in that CoA supposedly promotes binding of ATP and D-Ala, but subsequent release of CoA is required for the two to react. Presumably this means that the rate of the catalytic step for the adenylation reaction must be considerably faster than the rates of ATP and D-Ala dissociation, or else the enzyme could return to the apo state, and presumably the adenylation conformation, when CoA is released. Are these parameters known? Also, is the physiological concentration of DltC acceptor protein, which presumably takes over the role of CoA in vivo, high enough to be consistent with the proposed reaction cycle? It would need to rebind to the enzyme in the exchange step before the enzyme reverts to the adenylation conformation. Presumably the rate of DltC binding is faster than the rate of reversion back to the adenylation conformation? The authors should discuss/clarify these points.

Minor points:

 

1. The data in this paper demonstrate the effect on the enzyme by CoA, a mimic of the in vivo substrate DltC, but not for DltC itself. Is there some reason why this is difficult to demonstrate with DltC experimentally?
2. Was it previously known from other structures that the apo form of the enzyme is in the adenylation conformation, or is this a new insight? In general the authors could do a little bit of a better job describing the previous structures in the Introduction. Why were the previous structures of the *B. subtilis* enzyme found to be in the thiolation conformation despite the absence of CoA (or DltC)? Is there no structure available with CoA?
3.  References 16 and 18 are the same.

I have read this submission. I believe that I have an appropriate level of expertise to confirm that it is of an acceptable scientific standard.

# Response

**Published:** 2014-06-23

**Authors:** Yu Luo

**Affiliations:** Department of Biochemistry, University of Saskatchewan, Canada

As far as we know, the rate constants of substrate binding, reaction and product dissociation steps have not been measured for DltA or its closely related homologs that transfer the activated amino acyl or peptidyl group to a carrier protein. We do not have expertise in measuring such rate constants.

It is generally accepted that DltC is the D-alanine carrier *in vivo*. CoA, however, is a more feasible analog of DltC for enzymatic assays. If DltC were used, the assay would require either millimolar concentration of DltC or addition of a thioesterase to regenerate DltC, for which we haven't resolved the technical difficulties. 

The *B. subtilis *DltA in thiolation conformation has been co-crystallized with DltC, although DltC is disordered or absent from the crystal structure. Homologous acyl and aryl CoA synthetases have been known to crystallize in either adenylation or thiolation conformation. Remotely related Japanese firefly luciferase has been crystallized in an apo-form dissimilar to its adenylation conformation. However, such luciferases do not catalyze the second thiolation reaction and recently, DltA from *S. pyogenes* has been crystallized in the absence of any substrate. The structure (PDB entry 3L8C, to be published) resembles the adenylation conformation of *B. cereus* DltA.

# Peer review: Referee response for version 1

**DOI:** 10.5256/f1000research.4389.r5123

**Published:** 2014-06-16

**Authors:** Ting Fang Wang

**Affiliations:** 1 Institute of Molecular Biology, Academia Sinica, Taipei, Taiwan

This paper provides an intriguing two-conformation hypothesis for the enzymatic cycle of B. cereus D-alanyl carrier protein ligase DltA. Overall, both structural determination and biochemical assays were well done.

A minor point: The two-conformation hypothesis (Figure 6) suggests that the inclusion of ATP to the DltA-CoA-Ala complex functions includes CoA release and a large domain rotation. It is important to show that AMP-CPP, a non- hydrolyzable analog of ATP, can indeed induce CoA release.

I have read this submission. I believe that I have an appropriate level of expertise to confirm that it is of an acceptable scientific standard.

# Response

**Published:** 2014-06-23

**Authors:** Yu Luo

**Affiliations:** Department of Biochemistry, University of Saskatchewan, Canada

It would be interesting to carry out an experiment to study ATP-stimulated release of CoA or DltC. One plan is to use a surface plasmon resonance-based technique, or similar techniques, to study the association and dissociation of DltA with DltC under various conditions.
